# Traction-Assisted Endoscopic Submucosal Dissection of a Gastric Subepithelial Tumor: A Case Report

**DOI:** 10.7759/cureus.63649

**Published:** 2024-07-02

**Authors:** Anurag Sekra, Masato Yozu

**Affiliations:** 1 Department of Gastroenterology and Hepatology, Counties Manukau Health, Auckland, NZL; 2 Department of Pathology, Counties Manukau Health, Auckland, NZL

**Keywords:** interventional endoscopy, subepithelial lesions, gastrointestinal stromal tumor (gist), traction, endoscopic submucosal dissection (esd)

## Abstract

Endoscopic submucosal dissection (ESD) is considered curative for patients with early gastrointestinal cancers. However, it is a technically challenging procedure that can be time-consuming and associated with complications such as bleeding and perforation. Traction devices and techniques have been developed to mitigate these risks and reduce procedure times. Most traction devices are unavailable in New Zealand, and traction techniques have not been widely utilized due to the precision required for successful outcomes.

We report the first case of traction-assisted ESD performed in New Zealand for a gastric submucosal tumor. The procedure was successfully performed using the clip with rubber band traction technique. The lesion was resected en bloc, and histology confirmed an R0, curative resection. There were no complications, and the total procedure time was 54 minutes. In conclusion, traction techniques can be effectively employed for ESD in lesions with difficult submucosal access. They contribute to safer dissections and reduced procedure times.

## Introduction

Endoscopic submucosal dissection (ESD) is widely practiced in Asia for resecting early gastrointestinal (GI) cancers and submucosal tumors. Despite being less invasive than surgery, ESD presents technical challenges such as prolonged procedure times and increased risks of complications such as perforation and bleeding. Accessing the submucosal layer can be particularly difficult in lesions with fibrosis or when gravity cannot effectively provide counter traction, making ESD challenging and potentially hazardous in such cases. These challenges contribute to the limited availability of ESD in Western countries [1].

To address these issues, various traction devices and techniques have been developed over the years. Examples include the clip with line technique, clip and snare, clip-band technique, external forceps, double scope, magnetic anchor, and internal traction methods [2]. One innovative device, the S-O clip (Zeon Medical), introduced in Japan in 2016 initially for colorectal ESD, has since been extended to gastric and duodenal ESD [3].

Traction devices enhance visualization of the submucosal layer, enabling precise identification of the cutting plane. This not only reduces procedure times and complications but also encourages wider adoption of ESD in Western medical practices. A recent meta-analysis demonstrated that traction techniques are associated with shorter procedure times, higher rates of R0 resection, and lower risks of perforation [4].

Proximal gastric body lesions pose specific challenges during ESD due to significant looping in the forward view and difficulty accessing the oral side in the retroflexion view. This complexity makes the procedure risky, time-consuming, and challenging to perform [5]. One effective approach to overcome these challenges is using traction devices. However, most traction devices are not currently available in New Zealand (NZ). Other traction methods, such as the clip with line technique or clip with rubber band technique, which require precision, have not been utilized in NZ before. Herein, we present the first successful case of using the clip-band traction technique to facilitate ESD in NZ.

## Case presentation

**Figure 1:**
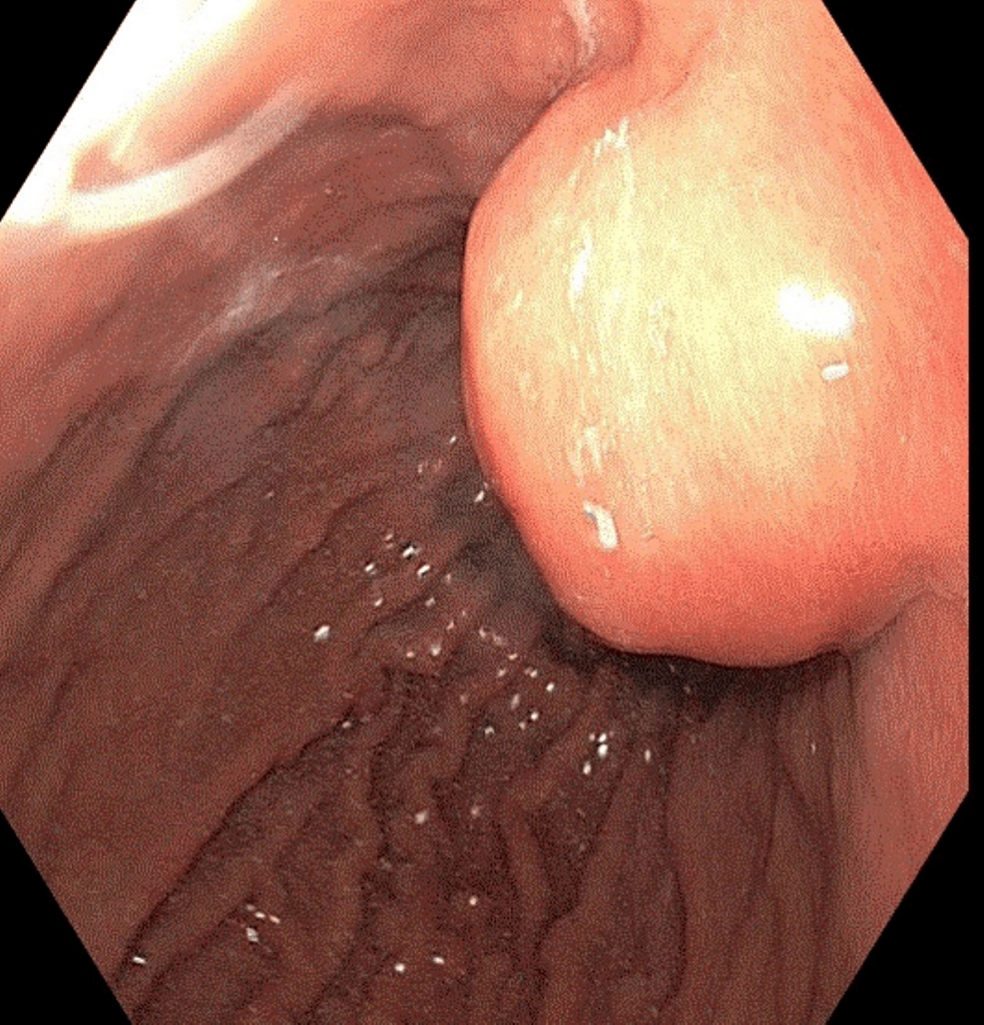
A 3 cm submucosal lesion in the gastric body along a lesser curve on routine endoscopy

An 81-year-old NZ European female underwent an upper GI endoscopy due to iron deficiency anemia. The procedure identified a 3-cm submucosal lesion located at the proximal gastric body along the lesser curvature of the stomach (Figure 1). Subsequent endoscopic ultrasound (EUS) revealed a 3 cm hypoechoic lesion arising from layer 3 (submucosal layer), with a possible attachment to layer 4 (muscularis propria). These findings were consistent with a lesion resembling a GI stromal tumor (GIST). Fine-needle aspiration performed during EUS yielded inconclusive results. A computed tomography (CT) scan did not indicate any extraluminal extension of the lesion.

Her case was discussed in the upper GI multidisciplinary meeting (MDM), and the consensus was to perform an endoscopic submucosal dissection (ESD) for a definitive diagnosis with curative intent. The patient consented to the procedure, and the risks and benefits were discussed.

Endoscopic submucosal dissection (ESD) procedure

**Figure 2:**
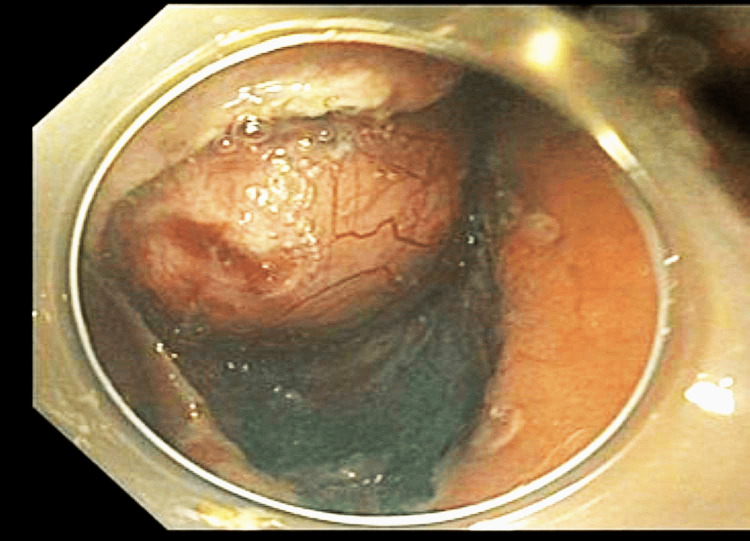
Tumor exposed at the oral side of the lesion

The procedure was conducted under general anesthesia. A standard gastroscope (Olympus GIF-HQ190, Olympus, Tokyo, Japan) equipped with a transparent hood (Olympus, Tokyo, Japan) was utilized. Submucosal lifting was achieved using a combination of adrenaline and Everlift solution (Everlift®, USA). A 2-mm dual knife J (Olympus, America) in endocut mode on the ERBE VIO 300D (ERBE, Elektromedizin, Tubingen, Germany) was employed. The periphery of the lesion was marked circumferentially using forced coagulation. A mucosal incision was initiated from the oral side of the lesion. Subsequent trimming of the submucosa and dissection proceeded until the tumor was fully exposed (Figure 2).

Further dissection under the tumor was performed until it could be done safely. The mucosal incision was then extended orally, and a circumferential mucosal incision was completed. Attempts were made to trim and dissect the submucosa from the anal side in both forward and retroflexion views of the endoscope. However, due to significant looping, access to the further submucosal plane was challenging, and consequently, dissection could not be continued safely.

**Figure 3:**
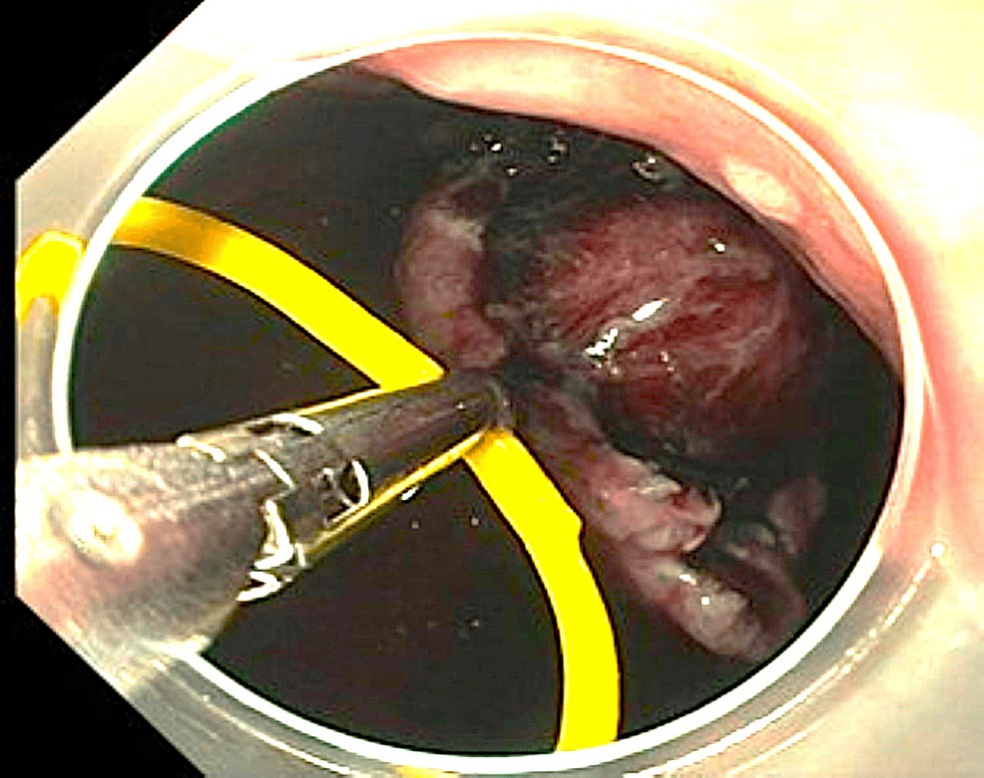
Clip-Band traction applied at mucosal flap from the oral side

**Figure 4:**
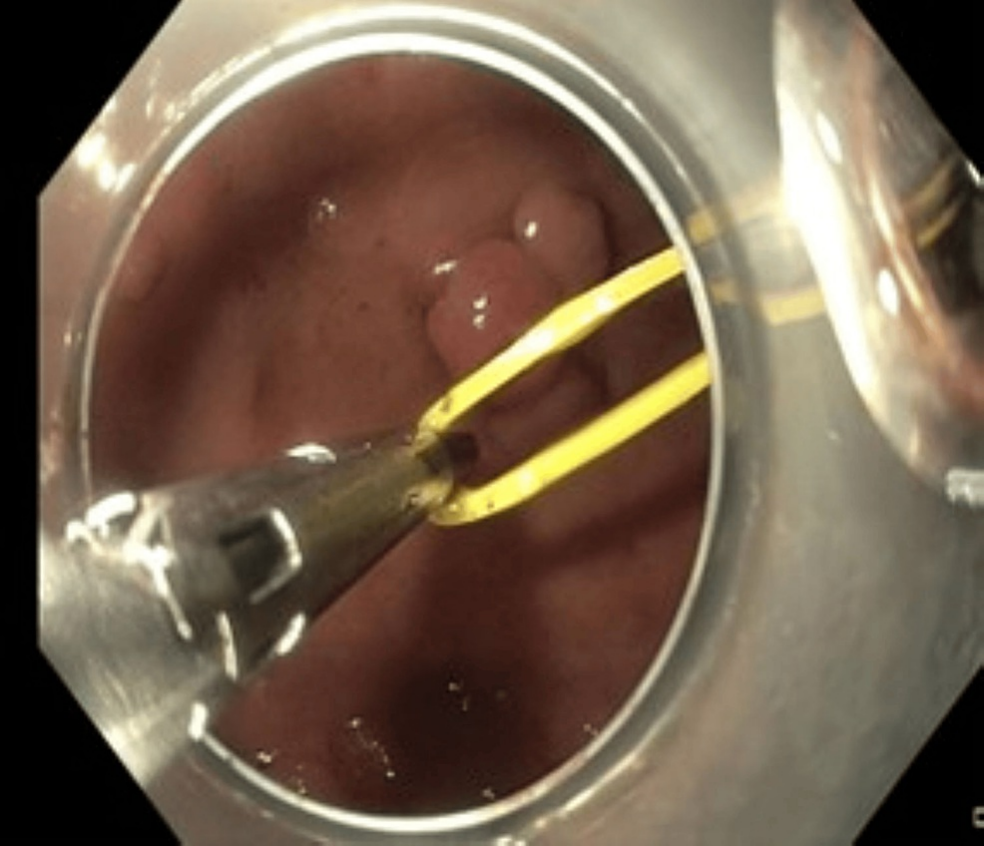
The other side of the band was captured with a second clip and deployed in the gastric fundus creating counter-traction

**Figure 5:**
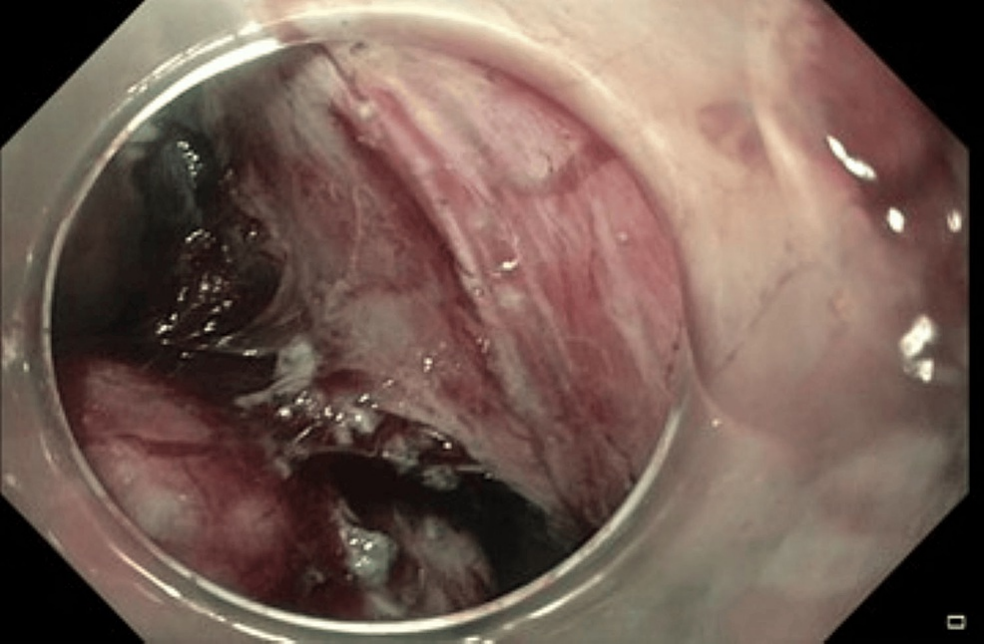
Access to submucosal space established after applying clip-band traction The counter traction made dissection safe and fast.

At this point, we decided to apply the clip-band traction technique to gain submucosal access. We preferred this traction technique due to our previous experience with it. We used a Resolution clip (Boston Scientific, USA), and a rubber band was attached to the clip. The clip was deployed at the mucosal flap of the lesion on the oral side (Figure 3). The other side of the rubber band was captured using another Resolution clip and deployed in the gastric fundus to provide countertraction (Figure 4). This provided optimal access to the submucosal plane (Figure 5), and dissection was safely continued.

**Figure 6:**
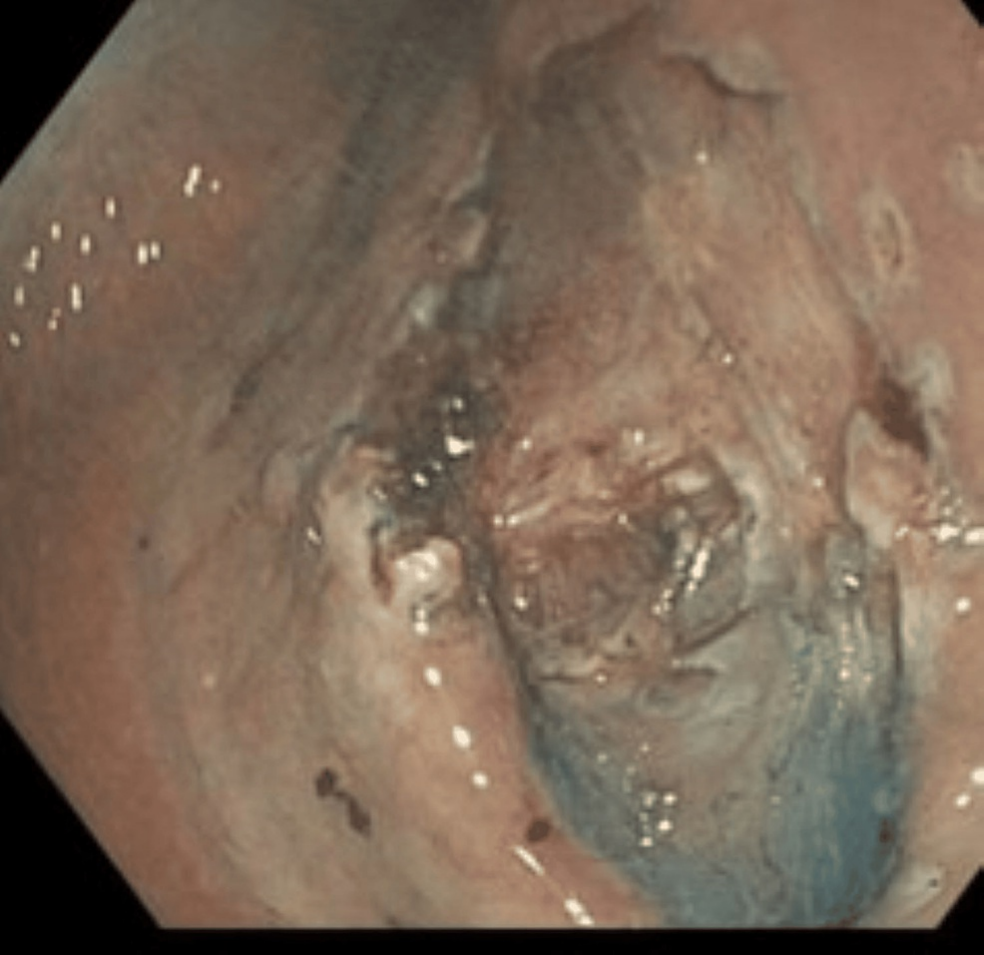
A small muscle defect after myotomy to release the tumor Circular myotomy was performed and longitudinal muscle was kept intact.

**Figure 7:**
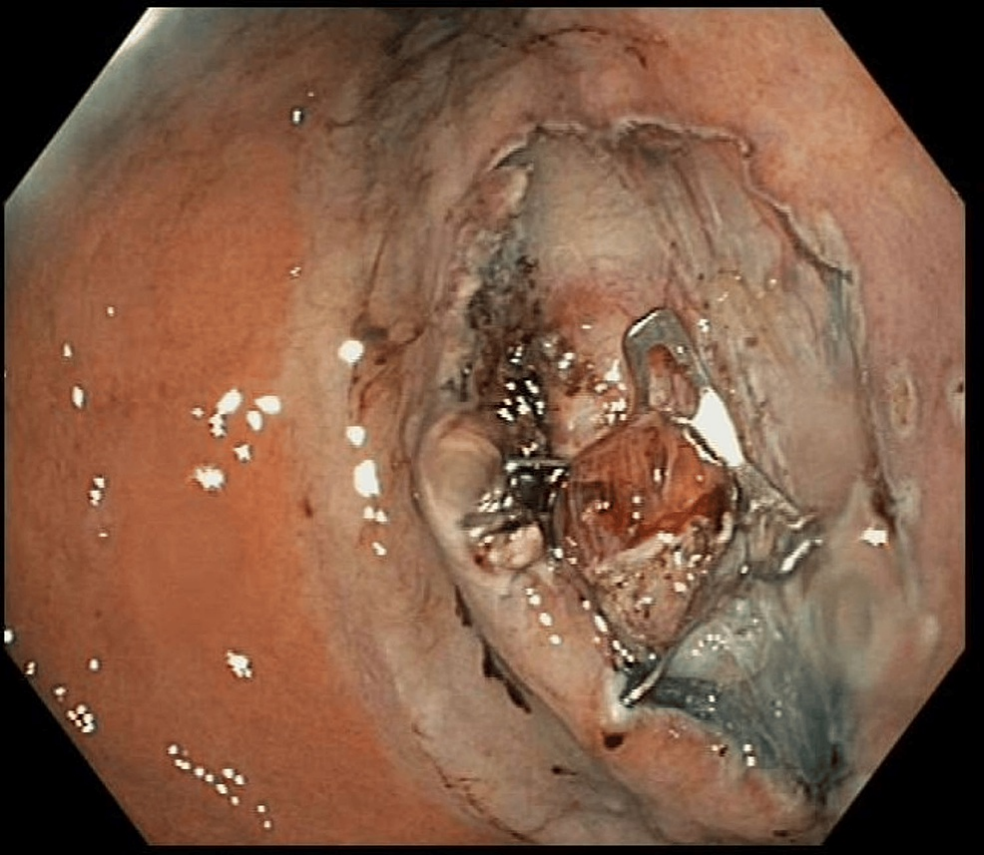
Myotomy defect was closed using the scope clip

**Figure 8:**
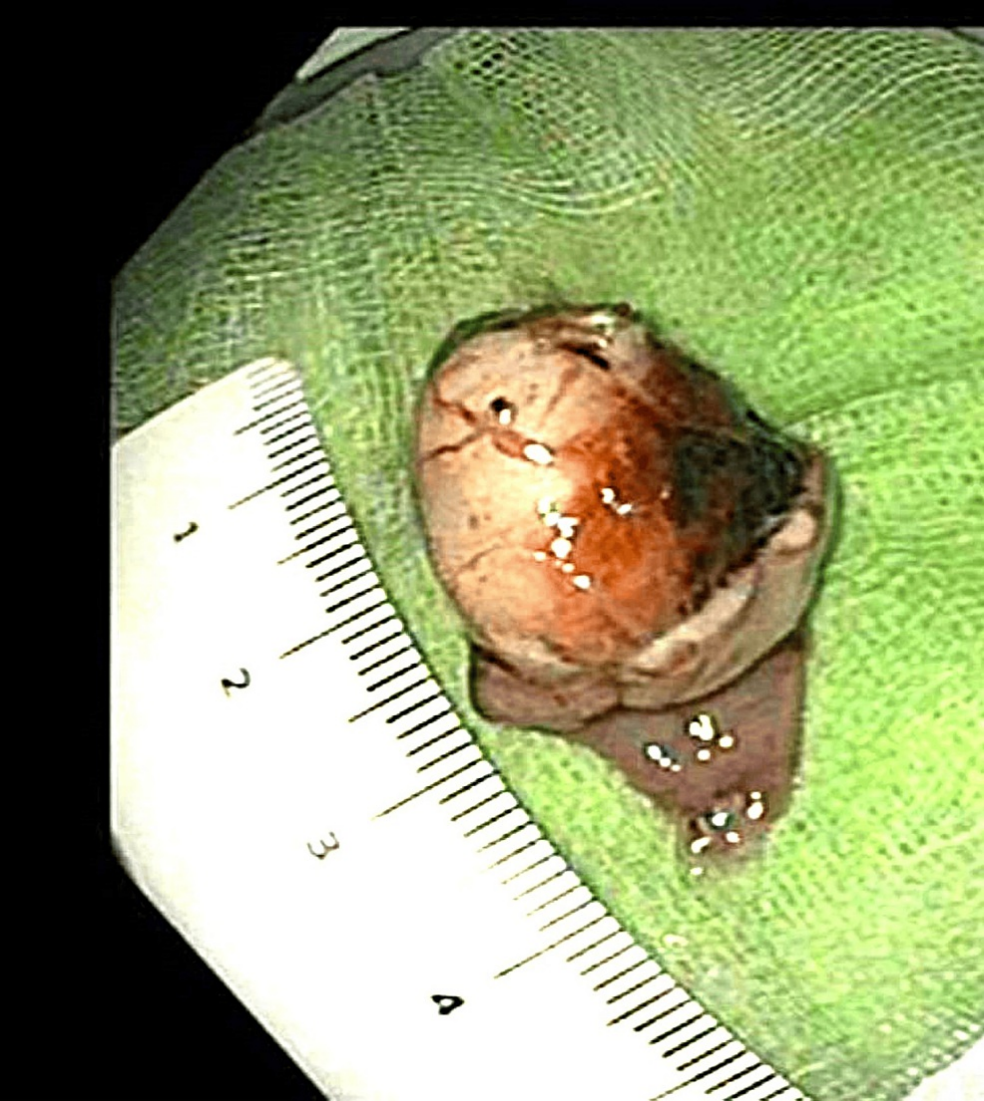
Lesion ex vivo with intact capsule

Further dissection under the tumor was performed, and an exceedingly small muscle attachment was identified at this stage. A myotomy was performed to release the tumor from the muscle layer. Due to the small size of the myotomy (Figure 6), a decision was made to close this area using an Over-The-Scope (OTSC) clip (Ovesco, Germany) (Figure 7). The lesion was successfully resected en bloc and retrieved for histology (Figure 8). The total procedure time was 54 minutes.

Histology

**Figure 9:**
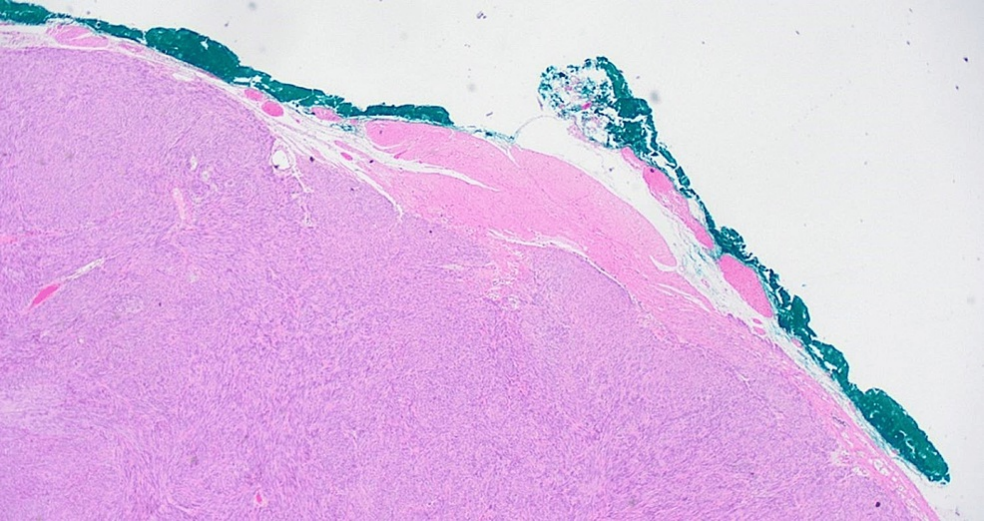
Low-grade gastrointestinal stromal tumor, pT2, negative radial and deep margins

Histologically, the specimen measured 31 mm × 26 mm and was covered by mucosa on one side, enclosing a well-circumscribed ovoid solid nodule measuring 25 mm × 18mm beneath the mucosa. Microscopic examination revealed that the nodule was predominantly composed of bland spindle cells with a very focal epithelioid component. Mitotic activity was low, with only one mitosis per 5 mm^2^, and there was no evidence of necrosis. Immunohistochemical analysis showed positivity for CD117 and DOG1, consistent with a low-grade GIST, pT2, with a very low risk of tumor progression (Figure 9). Both radial and deep margins were clear, confirming R0 resection.

Follow-up

The patient was admitted for observation and received intravenous antibiotics and clear oral fluids postoperatively. She remained well and was discharged on postoperative day 1. Her histology was reviewed in the MDM, which recommended a follow-up endoscopy in 3 months with no additional treatment required.

At the 1-year follow-up, the patient remained asymptomatic, and a repeat upper GI endoscopy for surveillance revealed no recurrence of the lesion.

## Discussion

ESD is an organ-preserving surgery performed for en-bloc resection of early GI cancers with curative intent [6]. Originating in Japan in the 1990s, ESD is increasingly adopted in Western countries for managing early GI cancers [7]. It enables en-bloc resection with higher rates of R0 resection (clear vertical and radial margins histopathologically), achieving curative treatment without the need for radical surgery or additional therapies [8].

The alternative methods include endoscopic mucosal resection (EMR) and radical surgery. EMR, while suitable for smaller lesions, does not facilitate en-bloc resection for lesions larger than 20mm due to the risk of perforation and technical challenges, often requiring piecemeal resection. This piecemeal approach complicates histopathological assessment and may necessitate additional surgery for confirmation of cure. [9]. EMR also exhibits higher rates of local recurrence compared to ESD [10] and recent cost analyses indicate that ESD is more cost-effective due to reduced need for surveillance colonoscopies [11].

Despite its advantages, ESD is a time-consuming procedure associated with higher risks of complications compared to EMR [12]. Advances in traction techniques and devices have improved the safety and efficiency of ESD procedures. However, the availability of traction devices remains limited in NZ, necessitating precise techniques for successful outcomes.

We successfully performed the first ESD using a clip-rubber band traction technique in NZ for a proximal gastric body lesion, where access to the lesion’s anal side was technically challenging in both forward and retroflexion views. The use of traction technique enhanced procedure efficiency, safety, and effectiveness, resulting in a successful outcome. The patient was discharged home on day 1, and histology confirmed R0 resection.

Despite its proven benefits, traction technique adoption in NZ is constrained by device availability. Our experience demonstrates that traction techniques can effectively provide access to the submucosal space for dissection in difficult cases, reducing procedural risks.

## Conclusions

Traction techniques offer a valuable tool for safe and efficient ESD, particularly in scenarios where gravity does not facilitate safe dissection. While currently underutilized in NZ due to limited device availability, increasing familiarity with these techniques and broader access to devices will likely enhance their acceptance and utilization.

## References

[1] Ma MX, Bourke MJ (2018). Endoscopic submucosal dissection in the West: Current status and future directions. Dig Endosc.

[2] Tsuji K, Yoshida N, Nakanishi H, Takemura K, Yamada S, Doyama H (2016). Recent traction methods for endoscopic submucosal dissection. World J Gastroenterol.

[3] Okamoto Y, Oka S, Tanaka S (2020). Clinical usefulness of the S-O clip during colorectal endoscopic submucosal dissection in difficult-to-access submucosal layer. Endosc Int Open.

[4] Lopimpisuth C, Simons M, Akshintala VS, Prasongdee K, Nanavati J, Ngamruengphong S (2022). Traction-assisted endoscopic submucosal dissection reduces procedure time and risk of serious adverse events: A systematic review and meta-analysis. Surg Endosc.

[5] Kim KO, Kim SJ, Kim TH, Park JJ (2011). Do you have what it takes for challenging endoscopic submucosal dissection cases?. World J Gastroenterol.

[6] Kakushima N, Fujishiro M (2008). Endoscopic submucosal dissection for gastrointestinal neoplasms. World J Gastroenterol.

[7] Friedel D, Stavropoulos SN (2018). Introduction of endoscopic submucosal dissection in the West. World J Gastrointest Endosc.

[8] Bhatt A, Abe S, Kumaravel A, Vargo J, Saito Y (2015). Indications and techniques for endoscopic submucosal dissection. Am J Gastroenterol.

[9] Draganov PV (2018). Endoscopic mucosal resection vs endoscopic submucosal dissection for colon polyps. Gastroenterol Hepatol (N Y).

[10] Rotermund C, Djinbachian R, Taghiakbari M, Enderle MD, Eickhoff A, von Renteln D (2022). Recurrence rates after endoscopic resection of large colorectal polyps: A systematic review and meta-analysis. World J Gastroenterol.

[11] Scheer S, Wallenhorst T, Albouys J (2022). Endoscopic submucosal dissection or piecemeal endoscopic mucosal resection for large superficial colorectal lesions: A cost effectiveness study. Clin Res Hepatol Gastroenterol.

[12] Strong A, Ponsky J (2016). Review: Endoscopic submucosal dissection (ESD) and endoscopic mucosal resection (EMR). Ann Laparosc Endosc Surg.

